# Human mate-choice copying is domain-general social learning

**DOI:** 10.1038/s41598-018-19770-8

**Published:** 2018-01-29

**Authors:** Sally E. Street, Thomas J. H. Morgan, Alex Thornton, Gillian R. Brown, Kevin N. Laland, Catharine P. Cross

**Affiliations:** 1 School of Biology, Sir Harold Mitchell Building, University of St Andrews, Greenside Place, St Andrews, KY16 9TJ Fife, UK; 2 Department of Anthropology, Durham University, South Road, Durham, DH1 3LE Country Durham, UK; 3 School of Human Evolution and Social Change, Arizona State University, South Cady Mall, Tempe, 85281 Arizona USA; 4 Centre for Ecology and Conservation, University of Exeter, Penryn Campus, Penryn, TR10 9FE Cornwall, UK; 5 School of Psychology and Neuroscience, University of St Andrews, Westburn Lane, St Andrews, KY16 9JP Fife, UK

## Abstract

Women appear to copy other women’s preferences for men’s faces. This ‘mate-choice copying’ is often taken as evidence of psychological adaptations for processing social information related to mate choice, for which facial information is assumed to be particularly salient. No experiment, however, has directly investigated whether women preferentially copy each other’s face preferences more than other preferences. Further, because prior experimental studies used artificial social information, the effect of real social information on attractiveness preferences is unknown. We collected attractiveness ratings of pictures of men’s faces, men’s hands, and abstract art given by heterosexual women, before and after they saw genuine social information gathered in real time from their peers. Ratings of faces were influenced by social information, but no more or less than were images of hands and abstract art. Our results suggest that evidence for domain-specific social learning mechanisms in humans is weaker than previously suggested.

## Introduction

The extent to which the decision making of animals is fine-tuned by natural selection in response to specific adaptive challenges or fashioned by general processes applying broadly across domains is a matter of ongoing debate^1,2^. Addressing this issue is central to the development of the fields of evolutionary psychology^1,3^, cognitive ecology^4^, and cultural evolution^5^, and human mate-choice copying represents a high-profile case in point. Evolutionary psychologists commonly argue that humans copy the apparent mate choices of others because doing so provides a selective advantage by reducing the search costs associated with finding a mate (e.g.^6–8^). The extent to which this process is underpinned by a domain-specific mechanism, however, is open to debate. It has been argued that humans select partners using domain-specific mental adaptations, specialised to process sexual cues^2,7,9^. Conversely, other work has applied theoretical work on domain-general social learning strategies to the specific question of mate choice^6,10^. Studies of mate-choice copying typically present heterosexual women with a photograph of a male face (hereafter a ‘target’) paired with one or more female faces (hereafter a ‘demonstrator’) and the participant is asked to rate the attractiveness of the target. The presence of the female faces is assumed to imply that the depicted females have selected the target as a mate, with facial cues commonly regarded as highly salient information in mate choice decision making. In such studies, an attractiveness rating given by a participant to a target is taken as a proxy for sexual interest in that individual.

By manipulating the conditions of mate-choice copying experiments, researchers have attempted to show that human mate-choice copying is attuned to the challenges of selecting a high-quality mate, when mate quality is largely determined by characteristics difficult to ascertain by personal observation alone. For example: participants’ ratings of attractiveness are influenced more strongly when they are asked to evaluate a target for a long-term, rather than a short-term, partnership^10,11^ (but see^12^). This is argued to be adaptive because a long-term partner’s suitability is based on qualities like prosociality and willingness to invest in offspring, which are not easily observable, but about which other individuals may have useful information^11^. Other studies have found that the preferences of attractive individuals are more likely to be copied than those of less attractive ones (reviewed in^10^). This is argued to be because, while most individuals form partnerships, only individuals of high mate quality are likely to have highly attractive partners: an attractive individual’s mate choices are therefore potentially the best indicator of mate quality (e.g.^11,13,14^).

While the above evidence suggests that mate-choice copying can produce adaptive outcomes for mate choice, it does not necessarily imply that the underlying mechanisms are domain-specific. Mathematical theory and experimental work both show that it is adaptive to copy others preferentially when asocial learning is costly or difficult, irrespective of the type of information about which a decision must be made^15–17^. The increased use of social information when evaluating a long-term partner might therefore reflect a general tendency to learn socially when the cost of a poor choice is high, or under conditions of uncertainty. Furthermore, a large body of literature shows that the preferences of prestigious individuals are more likely to be copied than those of less prestigious individuals – even if the information being copied is not related to the reasons for which the prestigious individual is highly regarded^18–20^. Accordingly, the aforementioned tendency preferentially to copy the mate preferences of attractive people – who are often assumed to be intelligent and prosocial^21^ – might reflect a general tendency to copy the choices of prestigious individuals, rather than a specific tendency to copy the mate choices of attractive same-sex others.

Although prior work has produced results consistent with a domain-specific mate-choice copying mechanism, in order to directly test for domain-specificity in mate-choice copying it is necessary to show that participants are more likely or more able to copy mate choices than other choices, all else being equal. Several studies have shown greater social influence on mate-choice decisions (i.e. an attractive same-sex individual choosing an opposite-sex target) than on non-mate-choice decisions (i.e. an attractive *opposite-sex* individual choosing a *same-sex* target)^6,10,14^. However, this experimental design confounds the choice to be made (i.e. the sex of the target) with the attributes of the source of the social information (e.g. the sex of the demonstrator). This means the apparent increase in social influence in a mate-choice context could result from observers placing more weight on the decisions of same-sex demonstrators than opposite-sex demonstrators, perhaps because they see the information offered by same-sex demonstrators as more personally relevant. Other studies do not contain this confound, but only manipulate the attributes of the demonstrator (e.g. apparent sex, facial expression) and hold the attributes of the target constant^10^. Therefore, it remains unknown whether humans show preferential social learning for attractiveness of a potential mate as opposed to other, more general, judgements of image attractiveness. Here, therefore, we directly tested the domain-specificity of mate-choice copying. We tested the hypothesis, derived from the evolutionary psychology work discussed above, that social influence on facial preferences differs from social influence on other preferences, when the source of the social information (i.e. the demonstrators) is held constant.

**Figure 1:**
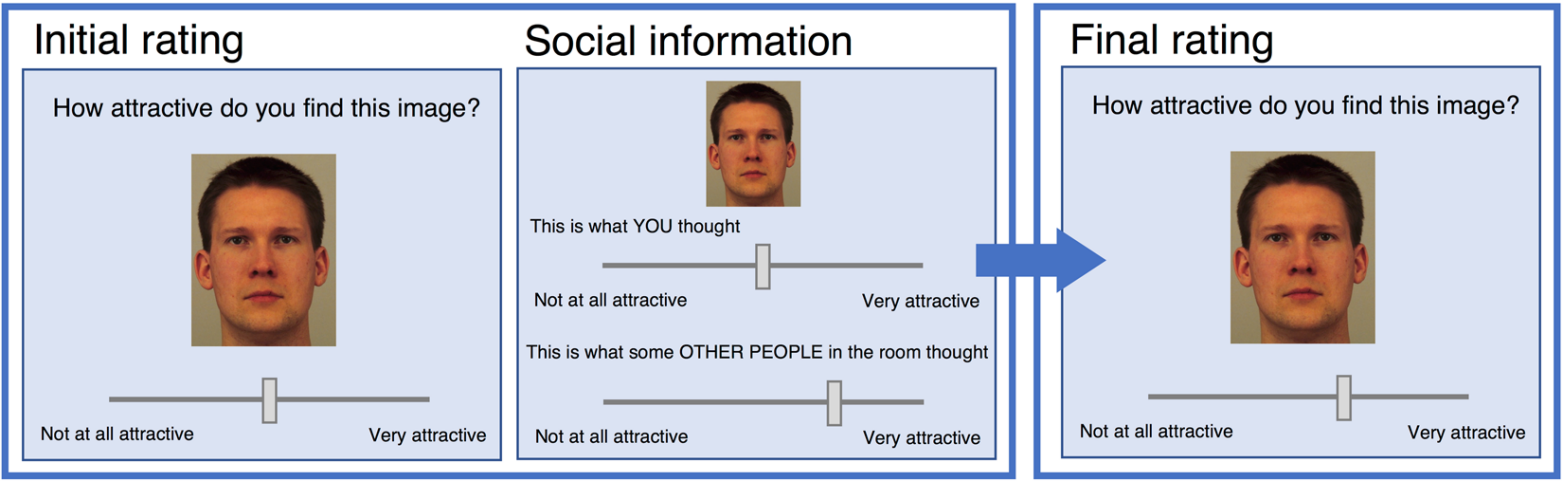
Schematic of the experimental task. Participants provided initial ratings and then viewed social information immediately afterwards, for each image within a block. After finishing a block of initial ratings and social information, participants viewed the same images again in a different random order and provided final ratings.

In contrast to previous experimental studies, we used real social information gathered from other participants. While artificially-generated social information allows greater experimental control, false information may be identified as such by participants during the experiment, which makes it difficult to interpret participants’ behaviour in response to it^22^. Further, deception creates ethical issues which affect future research^23^. We presented groups of female participants with pictures of male faces (the standard image type in mate-choice copying experiments), male hands (which are not commonly used in such experiments, but which allow a judgement of non-facial opposite-sex attractiveness to be made), and abstract art (which allow attractiveness judgements outside of the domain of mate-choice). In each case, participants answered the question ‘How attractive do you find this image?’ before seeing genuine social information (Fig. 1)- the average rating given by some or all of the rest of the group: this design enabled us to increase the variance in the social information seen by participants without using deception. Subsequently, after a short delay, participants re-rated each image. We used a Bayesian analysis to model participants’ final ratings using initial ratings, social information, and a social influence parameter which was estimated from the data for faces, hands, and artwork separately. This allowed us to evaluate whether the estimates for social influence differed across image types. The model also included a random effect for individual participants (see Methods).

**Figure 2:**
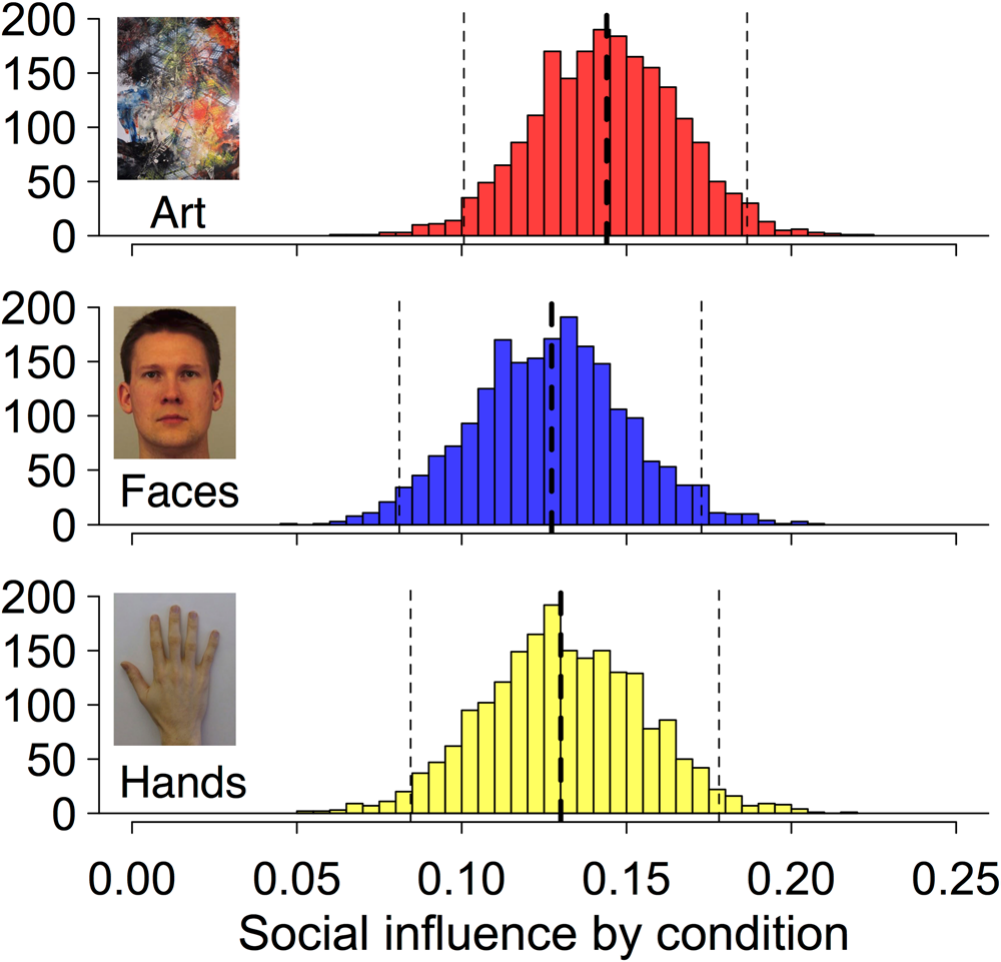
Histograms showing posterior distributions summarised across three chains for the social influence parameter estimated separately by each type of image: abstract artwork, faces, and hands. Heavy dotted lines represent the estimated median; light dotted lines represent 95% credible intervals. Artwork shown is by Waldemar Smolarek, CC BY-SA 3.0 (https://creativecommons.org/licenses/by-sa/3.0/). For the original, see https://commons.wikimedia.org/wiki/File:Abstract_oil_on_paper_w._smolarek_242.JPG. Face image shown is AM11NES from The KDEF^33^.

We found that social information did indeed affect participants’ final ratings of images of faces: for every point that the social information differed from their own rating, participants adjusted their rating by 0.13 points towards the social information (social influence parameter median estimate for faces = 0.13, 95% CI: [0.04, 0.21]). In other words, on average, a participant changed from their initial rating around 13% of the way towards the social rating, when rating the attractiveness of faces. We found highly similar effects, however, for images of hands (social influence = 0.13, [0.04, 0.22]) and abstract art (social influence = 0.14, [0.06, 0.23] (Fig. 2)). Contrasts between the social information parameters for the different stimulus types find no evidence of substantive differences and in fact provide strong evidence that the differences are close to, if not precisely, 0 (faces – hands =  <−0.01 [−0.06, 0.05], faces – art = −0.01 [−0.06, 0.03], art–hands = 0.01, [−0.03, 0.06]).

As with other experimental studies of social learning^17^, we can only infer that social information has been used because participants have already committed to an individual decision, which they report being able to remember (SI 1.3). Despite this, we have strong evidence that participants’ final ratings are influenced by social information (the CI for all three social influence parameters excludes zero). Furthermore, there was very little evidence of consistent individual differences in copying (variance of random participant effect = 0.06 [0.04, 0.10]). These results are concordant with the self-reports of our participants, half of whom reported that they used a combination of their own opinion and social information when rating images, while the remainder reported using ‘mostly or only’ their own judgement (SI 1.3). Naïve participants would be expected to use social information to a much greater extent, although quantifying the effect of social information on naïve participants in experimental studies is methodologically challenging. The majority of our participants reported not knowing any other members of the group (SI 1.3), and there were no immediate costs to ‘poor’ decisions in our experiment. It is possible, therefore, that the influence of social information observed by us underestimates the reliance on social information ‘in the wild’, where the decisions of close social relations are likely far more salient, and the consequences of ‘poor’ decisions far more costly.

Our study, which used an innovative experimental design providing genuine, real-time social information, thereby avoiding the deception of participants, found evidence of mate-choice copying in the sense that attractiveness ratings of faces were subject to social influence. Nonetheless, our findings suggest that it is unnecessary to propose either a face-specific, or a mate-choice specific, mechanism for this finding, because the social influence on facial preferences was no different from that on hands or abstract artwork when the source of the social information remained constant. This implies that what is widely referred to as ‘mate-choice copying’ in humans is most likely the product of domain-general social learning mechanisms that operate across a wide range of decision types. Previous studies demonstrate that human social learning is not applied inflexibly: individuals can strategically increase reliance on social information depending on contextual cues (e.g., high uncertainty^24^, high cost of error^16^, information from prestigious demonstrators^18^). However, making a choice specifically about potential mates does not, in itself, increase reliance on social information. While factors that influence social learning might well produce adaptive behaviour with regard to mate choice^10^, the available evidence provides little support for an evolved psychological adaptation selected specifically to process information relevant to mate choice.

Previous studies showing social influence on mate preferences in humans have explained these effects with reference to selective advantages specifically in the domain of mate choice^6,11,14^. None of these studies, however, manipulated the type of decision to be made (i.e. mate choice vs not mate choice) while providing genuine social information from the same group of conspecifics, as we have done here. As such, existing work cannot provide strong evidence for domain-specific social learning. To use the terminology of the cultural evolution literature, content (in this case, whether the decision concerns mate choice or not) and context (in this case, whether the source of the information is the same or a different sex) have thus far been confounded, which means that a bias based specifically for mate-choice content cannot be inferred^25^. It should also be noted that previous studies asked participants for their sexual orientation at the beginning of the study (rather than at the end, as in our study), which could have made decisions involving heterosexual partner choice particularly salient to participants, thereby increasing their attention to social information in those conditions. It may be that the use of social information increases for opposite-sex faces, but not other types of stimuli, when participants are prompted to think about mating decisions. Crucially, however, this explanation would not require a domain-specific mate-choice copying mechanism. Finally, we note that while comparing social learning for same- and opposite-sex faces within the same participants would have been an equally valid test of domain specificity, other lab-based work on conformity has already shown social influence within female participants for attractiveness ratings of female faces^26^.

A potential counter-argument to our interpretation is that decisions underpinned by domain-specific adaptations may in practice ‘bleed over’ to influence other types of decision^27,28^. For instance, even highly domain-specific facial processing mechanisms are argued to be co-opted for other types of images^29^. According to this view, although our results indicate that social influence is similar for different stimuli, it could nonetheless have been initially selected for because of the advantage conferred on individuals who copied favourable mate choices. In order to make this argument, however, evidence would need to be provided that copying of mate choices - rather than copying in any other domain (e.g. food choices) - was the relevant selective advantage. Costly and difficult decisions are widespread and not confined to mate choice. In all human societies, for instance, decisions must be made about where, when and how to shelter, forage, hunt, manufacture and use tools, and participate in social activities, with potentially severe consequences if poor choices are made. The adaptive value of copying in domains other than mate choice is well supported by existing literature^15–17^. Domain-general learning – in this case, from social information – is the most compelling explanation for our results.

Our study, like many others in the human^6,7,13^ and animal^30^ literature, focuses on the choices of females. The reason for this focus is that previous work suggests that mate-choice copying is more likely to be adaptive for women than for men, because long-term ‘mate value’ is more difficult to assess in men^13^. Any content-specific social learning mechanisms for mate choice should, therefore, be particularly detectable in women. The fact that we found none in a sample of (heterosexual) female participants is therefore particularly relevant to previous literature. Because all of our participants were women, we could not test for sex differences. However, we also note here possible domain-general explanations which could account for a previous finding that social learning of mate preferences is more likely in women than men^8^ (but see^10–12,14^). First, women may use social information more than men in a range of circumstances because of lower confidence^24^. Second, lower consistency in women’s attractiveness ratings of male faces than men’s attractiveness in female faces^31^ might indicate that women’s preferences for facial images are less strongly held and therefore more amenable to social influence than men’s. The aetiologies of sex differences in confidence and sex differences in consistency of attractiveness judgements are themselves open questions and the relative importance of evolutionary history, cultural norms, and interactions between the two are yet to be elucidated.

We emphasize that our findings do not contradict the idea that social learning of mate preferences takes place, nor that mate-choice copying has important evolutionary consequences^32^, nor that it can produce adaptive behaviour^10^. Rather, our results help to identify the processes by which mate-choice copying operates, and better inform future hypotheses and predictions regarding its function and evolutionary origin. More generally, our results caution against describing and interpreting behaviours as being specified for particular domains until alternative explanations have been ruled out^1,3^. Future investigations of how social learning strategies operate across tasks manipulating both content and context independently would be of great value in furthering our understanding of when, and why, we copy.

## Methods

### Participants

Forty-nine female participants were recruited from the University of St Andrews’ Participant Pool for a 30-minute experiment “Image Preference Game”. We used a neutral title for our experiment in study advertisements to reduce demand characteristics. The majority of participants were undergraduate students. Each received a £5 Amazon voucher for participation. Participants completed the study in 6 groups of 5–10, using networked computers. While participants were aware of the other group members, they interacted directly only with their own computers, using monitor shields to ensure privacy of responses. The majority of participants did not know any of the other participants in their group (SI 1.3). Within each group, participants completed trials synchronously and the social information each participant observed came from the other participants in their group. Here, we analyse the data from the 42 participants who reported being (mostly or exclusively) heterosexual in the post-experiment questionnaire (SI 1.3). However, we find very similar results when we include non-heterosexual participants in our analysis (SI 2.1). All protocols within our study received ethical approval from the University Teaching and Research Ethics Committee of the University of St Andrews (approval code BL10116) and the study was carried out in accordance with this institution’s guidelines and regulations. All participants gave informed consent before commencing.

### Stimuli

Participants rated images of three different types: (1) images of male faces, (2) images of male hands and (3) images of abstract artwork. We used images of male faces following the conventional focus on faces as an attribute of potential mates in the existing literature. Though not conventional in the field, we used images of male hands to represent a non-facial attribute of potential mates, allowing us to draw comparisons in social influence between facial and non-facial attributes of mates. We used images of abstract artwork to represent a condition clearly unrelated to mate choice, as these images do not visually resemble realistic faces or human forms, while it is still possible to ask participants to rate attractiveness of all three image types, phrased in exactly the same way. Images of male faces (forward-facing, with neutral expressions) were obtained from two existing datasets, the Karolinska Directed Emotional Faces (KDEF)^33^ and the Fundação Educacional Inaciana (FEI) Face Database^34^. Images were edited only by cropping and by adjusting colour balance to increase similarity between the two datasets. The 20 images of male hands were obtained from photographs of volunteers from the University of St Andrews staff and student population. Right hands were photographed from above, against a white background. Images of hands were edited only by cropping and by adjusting colour balance to increase similarity across the dataset. Images of abstract artwork were obtained from Wikimedia Commons.

### Procedure

After the experimenter read the instructions aloud (SI 1.2), participants were given the opportunity to ask questions (none did) before completing the experiment, followed by a short questionnaire (SI 1.3). Participants were asked not to speak to one another during the experiment (and none attempted to do so).

The experimental task was structured as either three blocks of 10 trials, or six blocks of five trials. This decision to use two different block lengths was made *a priori* in order to check that block length did not affect memorability of initial ratings. Because there was no effect of block length (SI 1.1), all trials are analysed together. Each block included a different, randomly selected subset of the stimulus images (a mixture of faces, hands and abstract art). Selection of images was balanced so that each participant rated an equal number of images of each type (faces, hands and artwork) across the task. Within each block (Fig. 1), the group progressed through the images in a random order rating the attractiveness of each image (‘initial ratings’) and then being randomly shown the average rating of some or all of their group members for that image (‘social information’). Once all images in the block had been rated, the group then progressed through the same images again (in a different randomly selected order) and were asked to re-rate the images for attractiveness (‘final ratings’).

For both initial and final ratings, participants gave their attractiveness ratings using a slider labelled from “not at all attractive” to “very attractive” with a hidden range of 0–100. Initially, the slider appeared in the centre of the scale, but participants could not proceed to the next screen until they had responded by clicking on the slider. The social information was shown on another such slider and randomly provided either (a) the mean of all other group members’ ratings, (b) the mean of the highest two group members’ ratings or (c) the mean of the lowest two group members’ ratings. This was done to increase the variation in social information without introducing deception. Participants were informed prior to the study that they would be shown the average of ‘some or all’ of the other participants (SI 1.2). Within the experiment, social information was always shown with the same wording (Fig. 1). No single individual’s ratings were identifiable to others (SI 1.2). All participant responses were self-paced, with response times automatically logged within the experiment. Participants took a mean of 6.92 (+/−3.78) seconds to provide an initial rating, 2.83 (+/−1.67) seconds to observe social learning information and 4.52 (+/−2.54) seconds to provide their final rating.

Each group completed either 3 blocks of 10 images (4/6 groups) or 6 blocks of 5 images (2/6 groups), corresponding to time delays of ~10 minutes and ~5 minutes between the initial and social ratings and the final ratings, respectively (this is similar to previous studies, in which delays of up to ~30 mins are used^26^). Participants’ self-reported memorability of initial ratings did not differ between blocks of different lengths (SI 1.1). We therefore pooled data across block lengths for all analyses. Each participant completed 30 trials, where each trial is a unique image seen and rated by each participant. The images used were drawn from larger sets (faces: N = 42, hands: N = 20, art: N = 20), however, so the same images were not necessarily used across groups of participants.

The post-experiment questionnaire (SI 1.3) included questions on how effectively participants were able to remember their initial ratings and what they perceived the intention of the experiment to be. When asked to report their perception of the intention of the experiment, no participants responded in a way to suggest that they misunderstood the task or did not believe that the social information was real (SI 1.3), indicating that our procedure for using real social information was perceived by participants as we intended it to be. Participants were also asked to report their sexual orientation using a 7-point scale, where 0 indicated exclusively heterosexual and 6 exclusively homosexual. For consistency with previous studies of mate-choice copying in humans^8,12,14^, we include only participants who self-identified as exclusively or near-exclusively heterosexual in our main analysis (N = 42, SI 1.3). We include non-heterosexual participants in a supplementary analysis, however, and results are qualitatively the same (SI 2.1).

### Analyses

We analysed the data using Bayesian GLMMs with Markov Chain Monte Carlo (MCMC) estimation in the R package rjags^35^. Prior to analyses, all ratings were transformed to fall between 0 and 1 using the formula Y = (X/100)*0.999 + 0.0005. Participants’ final ratings (N = 30 trials each from 42 participants = 1260 ratings) could therefore be treated as a beta distributed variable and modelled using a logit link function. We modelled final ratings as a function of initial ratings, social ratings, and a ‘social influence’ parameter, which estimates the extent to which participants’ final ratings were influenced by their own initial ratings versus the social information. Social influence of 0 corresponds to participants giving exactly the same rating before and after social information, whilst a value of 1 corresponds to participants abandoning their initial rating so that their second rating matches the social information exactly. Values between 0 and 1 indicate that the final rating was intermediate between the initial rating and the rating given in the social information, where values < 0.5 and >0.5 indicate a final rating closer to the initial rating or the social information respectively. Social influence values of >1 or <0 are also possible, and indicate ‘over-adjustment’ and ‘contrariness’ respectively.

We estimated the value of the social influence parameter separately for each condition (allowing social influence to vary according to the image type) with a random effect for each participant across conditions (allowing some participants to be more influenced than others). We effectively, therefore, fit a model with random slopes for image types and for participants. In a further analysis (SI 2.2), we show that allowing the random participant effect to differ between conditions does not alter the outcomes of the analysis. For all parameters, we report the median estimate and 95% central credible intervals.

Prior distributions for model parameters were as follows. The predicted final ratings were modelled using a beta distribution whose shape parameters were in turn estimated from the data and for which we used an exponential hyperprior with the rate parameter set to 1. We used normally distributed priors with mean 0 and variance 1 for the effect of image condition on social influence, and for the random participant effect. For the variance of the random participant effect, we used an exponential hyperprior with the rate parameter set to 1. For robustness, we carried out a second analysis with even flatter priors, obtaining highly similar results (SI 2.3). The model included 3 parallel chains and we confirmed convergence using the Gelman-Rubin statistic for each estimated parameter (all point estimates = 1, all upper C.I. s = 1). Each chain consisted of 50,000 iterations, with a ‘burn-in’ period of 5,000 iterations, thinning every 10 iterations to minimise autocorrelation, yielding effective sample sizes ranging from 2792 to 15957 for all parameters, combined across the three chains. All parameters are summarised across all three chains.

**Figure 3:**
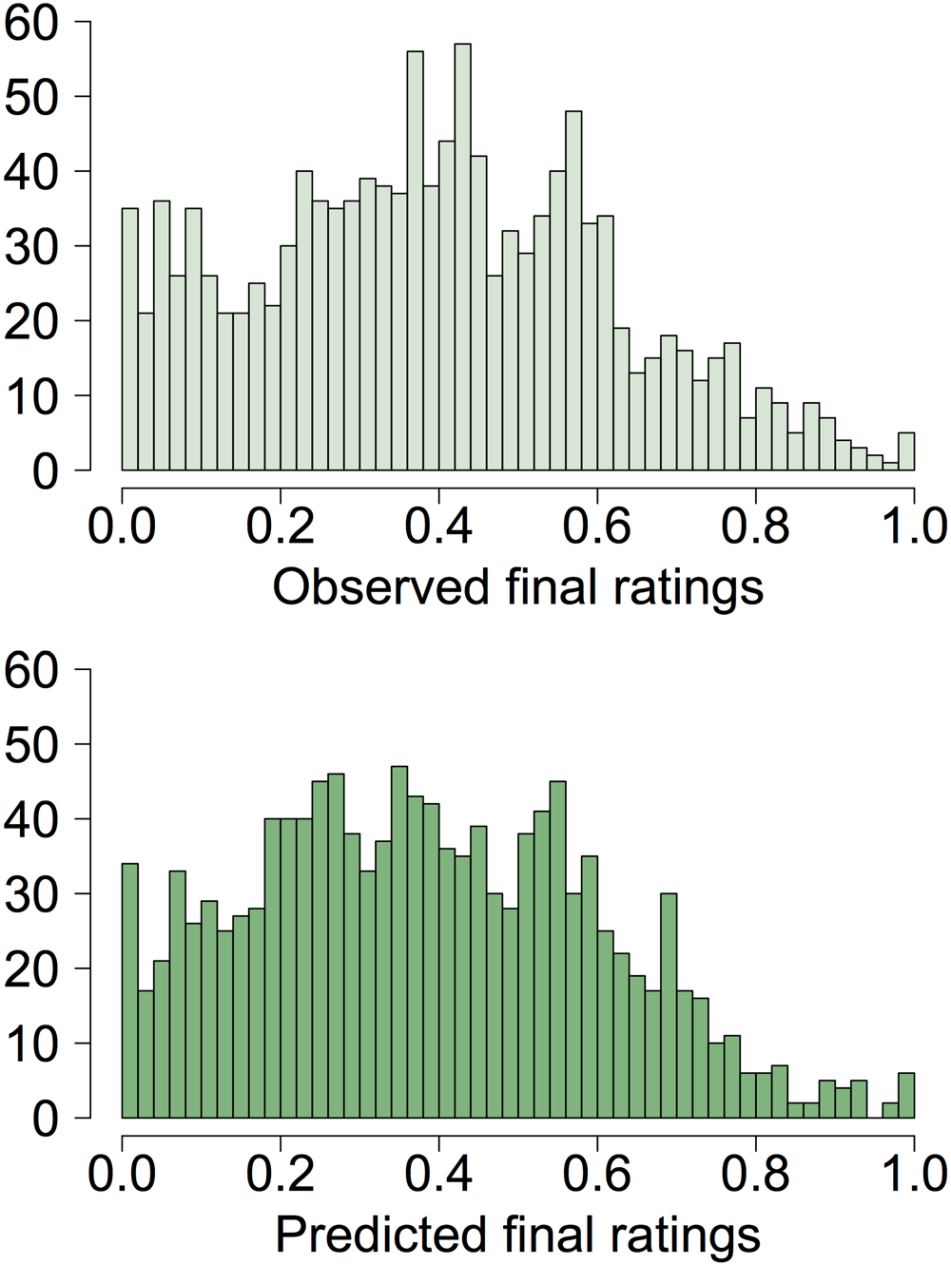
Histograms of observed final ratings and mean predicted final ratings from posterior distributions summarised across three chains.

Model performance was examined by visual comparison of predicted versus observed final ratings, and by estimating pseudo-R^2^ as the Pearson’s correlation of predicted and observed final ratings^36^. The distributions of predicted and observed final ratings were highly similar (Fig. 3), and the correlation between predicted and observed final ratings was 0.92 (pseudo-R^2^ 0.84, N = 1260), confirming that the model was appropriate for the data.

### Data availability

The dataset used in statistical analyses, all analysis code, and details of images used in the experiment, are available as supplementary material.

## Electronic supplementary material

Supplementary Information

Dataset 1

Dataset 2

Analysis code - main

Analysis code - supplementary
