# Supplementary material for: Human mate-choice copying is domain-general social learning
Source: Sci Rep. 2018 Jan 29;8:1715. doi: 10.1038/s41598-018-19770-8 (PMC5788917; doi:10.1038/s41598-018-19770-8)
Supplement: Supplementary file 1 — Supplementary Information [file 41598_2018_19770_MOESM1_ESM.pdf]

Supplementary Information:

Human mate-choice copying is domain-general social learning

Sally E. Street, Thomas J.H. Morgan, Alex Thornton, Gillian R. Brown, Kevin N. Laland, Catharine P. Cross

1. Experimental design

1.1 Memorability of initial ratings 2

1.2 Participant instructions 3

1.3 Post-experiment questionnaire 4

2. Supplementary Results

2.1 Supplementary analysis including non-heterosexual participants 6

2.2 Supplementary analysis allowing participant effects to differ between conditions 8

2.3 Supplementary analysis with flatter prior distributions 10

3. **Additional code**

3.1 STAN code supplied by an anonymous reviewer 12

1. Experimental design

1.1 Memorability of initial ratings

Our experimental design involved participants providing initial ratings and viewing social information for a block of images, before re-rating the images (‘final ratings’) following completion of the block (Methods). Participants completed the experiment in either in 3 blocks of 10 trials (for 4 of 6 experimental groups, N=33 participants) or 6 blocks of 5 trials (for 2 of 6 experimental groups, N=16 participants). Consequently, there was a time delay between providing initial ratings and providing final ratings, of ~10 minutes where longer blocks were used, or ~5 minutes where shorter blocks were used. Shorter blocks were used in addition to longer blocks, in case the greater time delay caused by longer blocks undermined the ability of participants to remember their initial ratings and social information when re-rating images.

In the post experiment questionnaire (SI 1.3), participants were asked to record, using a sliding scale, the extent to which they could remember their initial ratings when providing their final ratings, on a scale from 0 to 100. Participants reported mostly being able to remember their own ratings (mean 72.18, ±20.35). Further, self-reported memorability of initial ratings did not differ between participants who completed the study in 6 blocks of 5 trials (N=16) or 3 blocks of 10 trials (N=33, two sample T-test: T=-0.07, p=0.94). Therefore, we pooled data across the two blocking conditions for all analyses.

1.2 Participant instructions

Read aloud to participants:

“In this study you will see images of human faces, human hands and works of art. Your task will be to rate these images for attractiveness. You will receive a £5 Amazon voucher for taking part. The study consists of 30 questions arranged into [*3 blocks of 10/6 blocks of 5*]. Within each block, first you will rate all the images and be told what some of the other participants thought. You will be shown the average rating of some or all of the other participants, but you will not know whose ratings you are seeing. After rating all the images within the block, you will then re-rate all the images, although the order will be different. After you have completed all the blocks the study will end.”

“During the experiment, please interact only with the white window open on your screen now and please do not talk to other participants until you have been handed a debriefing sheet. You are free to withdraw at any time should you wish. One of us will be sitting in the adjacent room throughout the entire experiment should you have any problems.”

Ask if they all understand or have questions

“Please click the arrow on your screen to continue.”

1.3 Post-experiment questionnaire

After completion of the study, participants were asked to complete a short questionnaire. Participants were reminded that their responses were voluntary, and all questions included a ‘prefer not to say’ option. Participants had to be aged 18 or over to take part in the study, but we did not request participants’ ages in the post-experiment questionnaire. Participants were asked to report their sexual orientation using a 7-point scale (where 0 indicated exclusively heterosexual and 6 exclusively homosexual). 42/49 participants reported their sexual orientation as 0 or 1, while 7/49 reported their sexual orientation as 2-6. Participants were asked to self-describe their ethnicity using a free response box. Participants were asked whether they did or did not know any other participants in the group. 15/49 reported that they did know one or more of the other participants in the group, 32/49 reported that they did not, and 2/49 reported ‘prefer not to say’. Participants were asked to report, using a sliding scale, to what extent they could remember their initial ratings when providing their final ratings, where 100=maximum and 0=minimum, reporting a mean and standard deviation of 72.18, ±20.35.

Participants were asked to describe, using a free response box, how they decided to follow or ignore the social information they were shown. Of the 48/49 participants who responded to this question, 23 could be classified as reporting using a mixture of both their own judgement and the social information, while 24 reported using mostly their own judgement, and 1 gave an unclear answer. 15 of these 48 participants felt that they used the social information differently between the image types, and of 10 reporting being influenced most strongly by one image type in particular, 6 reported being most influenced for images of art, 4 for hands, and 2 for faces. Finally, participants were asked to describe, using a free response box, what they thought the intention of the experiment was. Of the 48/49 participants that responded to this question, all but one understood that the point of the experiment was to study social learning, but only 3 showed awareness that the intention was

to compare copying between different types of image. No participant responded in a way suggesting that they misunderstood the experimental task, or did not believe that the social information was genuine. Therefore, we were confident that participants understood the experimental task and treated the social information as genuine.

2. Supplementary results

2.1 Supplementary analysis including non-heterosexual participants

In our main analysis, we include only those participants (N=42) self-identifying as exclusively or near-exclusively heterosexual (0 or 1 on a 7-point scale where 0 indicates exclusively heterosexual, and 6 exclusively homosexual). When running our analysis on all (N=49) participants, we find highly similar results.

Model performance

As in our main analysis, there was a correlation of 0.92 between predicted and observed final ratings (pseudo-R^2 0.84, N=1470), confirming that the model was appropriate for the data.

Chain performance

Similarly to our main analysis, chain convergence was confirmed by large effective sample sizes (range 3472 to 16538), and Gelman-Rubin statistics (all point estimates = 1, all upper C.I.s = 1.01) across all estimated parameters.

Effect of condition on social influence

Similarly to our main analysis, when all participants were included, social information affected participants' final ratings of images of faces (social influence median estimate = 0.14, [95% CI: 0.06, 0.22]), hands (social influence = 0.12, [0.04, 0.19]) and abstract art (social influence = 0.15, [0.08, 0.22]). Again, medians and 95% CI for contrasts in social influence between conditions suggested that differences were very close to zero (faces – hands = 0.03 [-0.02, 0.07], faces – art = -0.01 [-0.05, 0.04], art – hands = 0.03, [-0.02, 0.08]).

Individual participant effects

As in our main analysis, the median variance of the random participant effect was 0.06 [0.04, 0.09], suggesting that relatively little of the variance in social influence was explained by consistent differences in social influence between participants.

2.2 Supplementary analysis allowing participant effects to differ between conditions

As in our main analysis, here we include only those participants (N=42) self-identifying as exclusively or near-exclusively heterosexual.

Model performance

Very similarly to our main analysis, there was a correlation of 0.92 between predicted and observed final ratings (pseudo-R^2 0.85, N=1260), confirming that the model was appropriate for the data.

Chain performance

The model included three parallel chains, each of 50,000 iterations thinned by 10 to reduce autocorrelation. At completion, the effective sample size for the different variables ranged from 4,813 to 16,068. Chain convergence was checked using the Gelman-Rubin convergence diagnostic (all point estimates = 1, all upper C.I. = 1, except in one case where the upper C.I. was 1.01).

Effect of condition on social influence

Very similarly to our main analysis, when allowing participant effects to vary with condition, social information affected participants' final ratings of images of faces (social influence median estimate = 0.13, [95% CI: 0.04, 0.22]), hands (social influence = 0.15, [0.05, 0.25]) and abstract art (social influence = 0.13, [0.04, 0.22]). Again, 95% CI for contrasts in social influence between conditions suggested that differences were close to, if not precisely, 0 (faces – hands = -0.02 [-0.15, 0.12], faces – art = <0.01 [-0.13, 0.13], art – hands = -0.02, [-0.15, 0.11]).

Effect of condition on participant effects

We find that the variance between participants in social influence is very similar for images of artwork (0.08 [0.05, 0.12]), faces (0.08 [0.05, 0.12]) and hands (0.08, [0.05, 0.14]). All contrasts between conditions in the between-participant variance were very close to zero (faces – hands = -0.01 [-0.06, 0.05], faces – art = >-0.01 [-0.05, 0.05], art – hands = -0.01 [-0.07, 0.05]).

2.3 Supplementary analysis with flatter prior distributions

The careful choice of priors is an essential part of a Bayesian analysis. In the main paper, we present the results of an analysis that used “weakly regularizing priors” as suggested by an anonymous reviewer. These are priors that minimally constrain the output of the analysis by encouraging the model to focus on biologically plausible values. For instance, the social influence parameters for each condition were given, as a prior, a normal distribution with a mean of 0 and a variance of 1. This encourages the model to favour estimates of these parameters with a magnitude close to 0 and gives a very high chance that the magnitude is less than 3. Given that a value of 0 is no social information use, and a value of 1 is total conformity, this seems reasonable. Nonetheless it is important to check that the results are not unduly influenced by the choice of priors, so here we present the results of another analysis in which the priors were extremely flat. In this case the priors for the condition effects are normal distributions with a mean of 0 and a variance of 100. The prior for the variation between participants, which was an exponential distribution with a parameter value of 1 in the main paper, is instead gamma distributed with a shape and rate of 0.001.

Weakly regularizing priors are typically considered a better option than these extremely flat priors, as our anonymous reviewer pointed out. For instance, the extremely broad priors for the condition effects imply that the conditions are likely to be extremely different from each other and characterized by extreme values of social influence. While biologically plausible values will mainly fall between 0 and 1, values such as -200 are treated as perfectly plausible by the model with flat priors. Nonetheless, with enough data the priors can be overwhelmed and results should not be unduly affected. We therefore present these additional results to verify the robustness of our findings.

Model performance

As with our main analysis, we found a correlation of 0.92 between predicted and observed final ratings (pseudo-R^2 0.84, N=1260), confirming that the model was appropriate for the data.

Chain performance

Chain convergence was confirmed by large effective sample sizes (range 5334 to 7068) and Gelman-Rubin statistics (all point estimates = 1, all upper C.I.s = 1.01).

Effect of condition on social influence

Replicating the results of our main analysis, social influence was highly similar for images of faces (median estimate = 0.13, 95% CI: [0.08, 0.17]), hands (0.13, [0.08, 0.18]) and abstract artwork (0.14, [0.10, 0.19]). As before, contrasts in social influence were effectively zero (faces – hands = <-0.01 [-0.05, 0.05], faces – art = -0.02 [-0.06, 0.03], art - hands = 0.01, [-0.04, 0.06])

Individual participant effects

Similarly to our main analysis, we found a low random participant effect (median estimate = 0.01 [<0.01, 0.02]), suggesting little evidence of consistent individual differences in social influence.

3. Additional code

3.1 STAN code supplied by an anonymous reviewer

The reviewer who suggested we use weakly regularizing priors also kindly provided code to run our analysis in STAN, another piece of Bayesian analysis software. STAN runs the same kinds of analyses as JAGS, but uses different sampling algorithms which can greatly increase the efficiency with which models run. If readers are familiar with both JAGS and STAN and wish to reproduce our analyses they will likely find that STAN is the faster way to do this.

```
# Stan model
library(rstan)

stan_model_code <- "
data{
int<lower=1> N;
int<lower=1> N_condition;
int<lower=1> N_player;
real final[N];
real initial[N];
real social[N];
int condition[N];
int player[N];
}
parameters{
vector[N_condition] b_condition;
vector[N_player] b_player;
real<lower=0> sigma;
real a;
```

```
real<lower=0> phi;
}
model{
vector[N] b;
vector[N] p;
phi ~ exponential( 1 );
a ~ normal( 0 , 1 );
sigma ~ exponential( 1 );
b_player ~ normal( 0 , sigma );
b_condition ~ normal( 0 , 1 );
for ( i in 1:N ) {
b = a + b_condition[condition] + b_player[player];
p = (1 - b) * logit(initial) + b * logit(social);
p = inv_logit(p);
}
final ~ beta( p*phi , (1-p)*phi );
}
generated quantities{
vector[3] social_influence_condition;
for ( i in 1:3 ) social_influence_condition = a + b_condition;
}
"

data_list <- list(
N=N,
N_condition=3,
N_player=N_players,
final=p.final,
```

```
initial=p.initial,
social=p.social,
condition=condition,
player=player
)

stan_fit <- stan( model_code=stan_model_code , data=data_list , chains=3 , cores=3 )

# diagostics and such
print(stan_fit,probs=c(0.025,0.975))

# extract permuted samples
post <- extract(stan_fit)
```
