# Supplementary material for: Human mate-choice copying is domain-general social learning
Source: Sci Rep. 2018 Jan 29;8:1715. doi: 10.1038/s41598-018-19770-8 (PMC5788917; doi:10.1038/s41598-018-19770-8)
Supplement: Supplementary file 3 — Dataset 2 [file 41598_2018_19770_MOESM3_ESM.pdf]

‘Street_et_al_image_preference_data_2017.csv’ contains the following columns:

**playerID**	numerical player identifier (1-49)
**trialID**	numerical trial identifier (1-1470)
**groupID**	numerical group identifier (1-6)
**nplayers**	number of players in group (5-10)
**condition**	type of image viewed and rated in the trial (art, faces or hands)
**questions.per.block**	number of questions in block (5 or 10)
**initial.rating**	initial attractiveness rating (minimum 0, maximum 100)
**initial.decision.time**	time taken to provide initial attractiveness rating (milliseconds)
**social.rating**	attractiveness rating of some or all other players (minimum 0, maximum 100)
**social.decision.time**	time taken to view social information (milliseconds)
**final.rating**	final attractiveness rating (minimum 0, maximum 100)
**final.decision.time**	time taken to provide final attractiveness rating (milliseconds)
**orientation**	participant sexual orientation (minimum 0=exclusively heterosexual, maximum 6=exclusively homosexual)
**know.anyone**	whether participant knew any others in the group (yes, no, prefer_not_to_answer)
**remember.initial.ratings**	to what extent participant reported being able to remember initial ratings when providing final ratings (minimum 0, maximum 100)
**use.social.info**	coded from free responses to question of how participant chose to use or ignore social information (mostly_individual = participant reported using only or primarily individual preferences, both_social_and_individual = participant reported using both social information and individual preferences, not_clear = participant did not provide a clear answer, no_answer = participant provided no answer).
**experiment.intent**	coded from free responses to question of what participant thought was the intention of the experiment (social_influence = participant perceived the intention of the study to be related to social influence, non_social_influence = participant perceived the intention of the study to be unrelated to social influence, social_influence_image_types = participant perceived the intention of the experiment as comparing social influence between image types, no_answer = participant provided no answer).
